# Adenovirus Core Proteins: Structure and Function

**DOI:** 10.3390/v13030388

**Published:** 2021-02-28

**Authors:** Shermila Kulanayake, Suresh K. Tikoo

**Affiliations:** 1 Vaccine and Infectious Disease Organization-International Vaccine Center (VIDO-InterVac), University of Saskatchewan, Saskatoon, SK S7N5E3, Canada; Shermila.kulanayake@usask.ca; 2 Vaccinology & Immunotherapeutics Program, School of Public Health, University of Saskatchewan, Saskatoon, SK S7N5E3, Canada

**Keywords:** adenovirus, nucleus, DNA genome, DNA packaging, core proteins, AdV protease cleavage

## Abstract

Adenoviruses have served as a model for investigating viral-cell interactions and discovering different cellular processes, such as RNA splicing and DNA replication. In addition, the development and evaluation of adenoviruses as the viral vectors for vaccination and gene therapy has led to detailed investigations about adenovirus biology, including the structure and function of the adenovirus encoded proteins. While the determination of the structure and function of the viral capsid proteins in adenovirus biology has been the subject of numerous reports, the last few years have seen increased interest in elucidating the structure and function of the adenovirus core proteins. Here, we provide a review of research about the structure and function of the adenovirus core proteins in adenovirus biology.

## 1. Introduction

**Figure 1:**
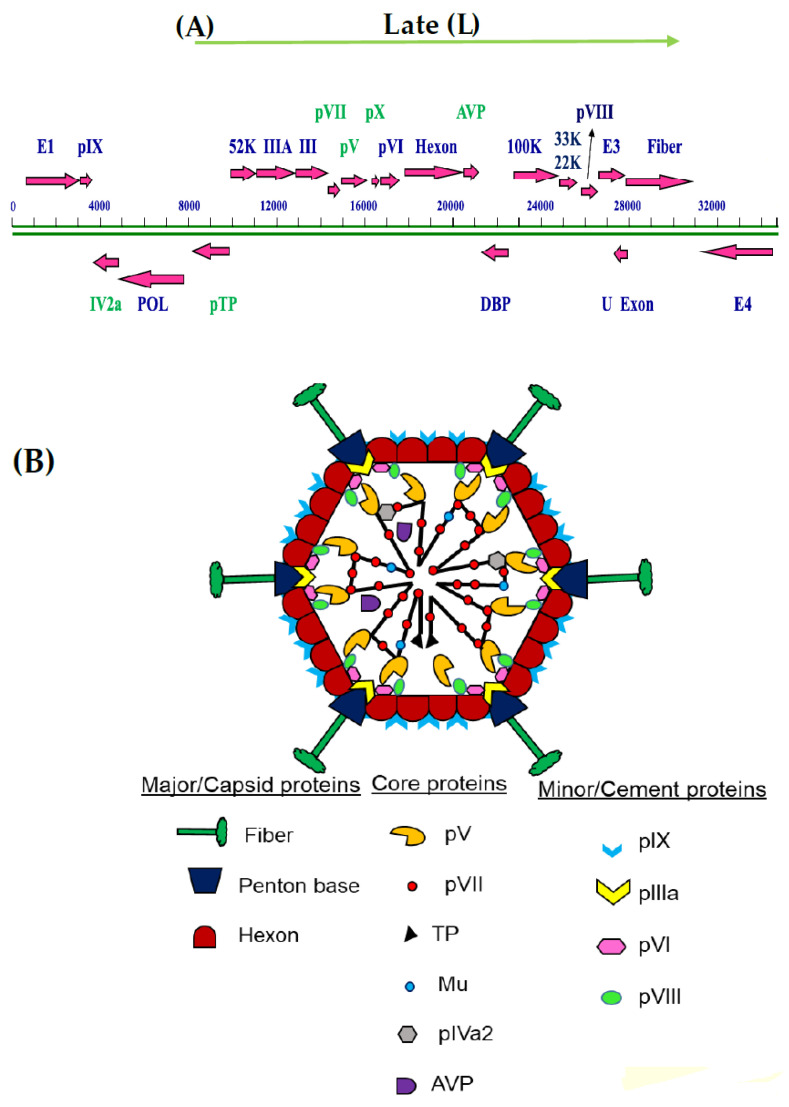
(**A**), Schematic diagram of the genome of *Mastadenovirus* (bovine adenovirus-3). Two lines show DNA strands. Numbers at the top show base pairs (BP). Arrows show the direction of transcription/translation. Core proteins are depicted in green. AVP—adenoviral protease; DBP—DNA-binding protein; POL—DNA polymerase; E—early; L—late. (**B**), Adenovirus structure: Schematic diagram of a cross-section of the adenovirus virion based on crystallography and cryo-electron microscopy. AVP—adenovirus protease; TP—terminal protease; p—protein. Adapted from ref. [1].

Adenoviruses (AdVs) are non-enveloped icosahedral viruses with a double-stranded DNA genome [1]. Since its discovery in 1953 [2,3], more than 120 species-specific adenoviral serotypes have been identified in humans, mammals, birds, fish and reptiles [4,5]. Though human adenoviruses are not generally associated with causing severe disease in immunocompetent humans, they may cause severe infections in immunocompromised people [6,7,8]. In contrast, animal adenoviruses appear to be associated with clinically important diseases in animals and birds [9,10]. AdVs, about 65–90 nm in size [1,11] with complex structural organization [11,12] have been grouped into six genera in Adenoviridae family [11,12]. Viruses in the Adenoviridae family contain a 26 to 48 kb [13] non-segmented linear double-stranded DNA genome [1,11], which transcribes at different times post-infection generating transcripts classified into early (E), intermediate (I) and late (L) regions [1,11]. The late (L) region encodes both structural and nonstructural proteins, which are involved in capsid formation, DNA encapsidation and maturation of progeny adenovirus virion (Figure 1A).

Members of *Mastadenovirus* (mAdV) genus contain proteins, which are genus-specific (protein IX and protein V) or shared (DNA polymerase (pol), terminal protein (TP), DNA-binding protein (DBP), 52K, protein (p) IVa2, pIIIa, pIII, pVII, pX, pVI, hexon, protease, 100K, 33K, pVIII and fiber) with members of other genera of Adenoviridae family [1,11] (Figure 1A). The mAdV virion capsid surrounds the viral core and is composed of major (penton, hexon, fiber) and minor (pIX, pIIIa, pVI and pVIII) [1,11] capsid proteins (Figure 1B), which play important roles in stabilizing the virion structure involving protein–protein interactions [1].

## 2. mAdV Core Proteins

The viral core of icosahedral DNA and RNA viruses consists of proteins associated with nucleic acids. The core of mAdV contains genomic DNA and core proteins pVII, pV, Mu(pX), pIVa2, terminal protein (TP) and AdV encoded protease (Figure 1) [1,11]. The terminal protein is attached to the 5’ termini of each genome segment and is involved in DNA replication [14]. The AdV protease binds non-specifically to genomic DNA and is involved in the cleavage of some adenovirus precursor proteins [15,16]. AdV IVa2, an intermediate protein, is considered a core protein because of its internal location in the virus particle [1]. Polypeptides VII, V, and X (Mu) are the major constituents of mAdV core nucleoprotein complexes [1] and are recognized as the major DNA condensing proteins responsible for about 50% of the core molecular weight [17,18]. They are rich in basic amino acids, which bring a positive charge, thus strongly binding to negatively charged dsDNA. This helps to condense the viral genome to fit within the limited space available inside the capsid [16,17,18], which appears crucial in (a) preventing recognition of the viral DNA by the host immune system and (b) maintaining the internal pressure of the virion and the release of DNA from the disrupted viral particles [18]. Mart´ın-Gonz´alez et al. explained the AdV core as a mixture of fibers and blob structures [18]. A model proposed based on cryo-electron tomography combined with molecular dynamics simulation analyses suggests that AdV core appears as several adenosome subunits (soft spheres, arranged like beads in a string with inter-adenosome DNA spaces, forming a thick fiber-like structure) composed of DNA and condensing proteins distributed asymmetrically [17].

In addition to playing important roles in the adenovirus life cycle, some viral core proteins have been used as tools to (a) deliver peptide vaccine antigens [19], (b) identify the intra-nuclear location for the accumulation of the viral capsid and core [20], and (c) quantitate adenovirus virions [21].

### 2.1. Protein VII (pVII)

#### 2.1.1. General Characteristics

The L2 region of mAdV encodes pVII [1,11], which is responsible for approximately 10% of the mass of the virion [22]. The N-terminus 50 amino acids of pVII orthologues encoded by members of mAdV appear highly conserved [23]. pVII is a highly basic protein rich in arginine (23%) and alanine (19%) residues. About 46% of pVII amino acids are positively charged [24] or highly cationic, which makes it attractive for the negatively charged phosphate backbone of DNA.

The secondary structure analysis of the protein [25] predicted two helix-loop-helix domains flanked by extended beta-sheets at both N- and C-termini of pVII. While the hydrophobic interactions between these helices allow pVII intra-chain folding, giving it a tertiary structure [26], the basic domains still interact with phosphates of genomic DNA exhibiting pVII as nucleosome-like particles composed of larger homo-oligomers in the adenovirus chromatin [26]. The pVII monomers are oligomerized before their nuclear import [26]. Interestingly, cryo-electron microscopy (cryo-EM) [27] analysis of AdV-5 (HAdV-5) at 3.2 A resolution suggested the presence of a cleaved pVII segment (amino acid 14 to 24) in the inner cavities of hexons, which was confirmed by mass spectrometry [28].

The tandem mass spectrometry analysis of pVII detected two acetylation sites on conserved lysine residues and three phosphorylation sites, which may be responsible for pVII interaction with the cellular chromatin and perform an important function during virus replication [29]. Interestingly, pVII is proteolytically cleaved apparently by the viral protease [15,30], recognizing a non-consensus cleavage site between residue 13–14 releasing peptide pVII_N1_ (N-terminus residues 1–13) and a consensus cleavage site (M/LXGG↓X) [15,31] between residue 24 and 25 releasing peptide pVII_N2_ (N-terminus residues 14–24 are released) [15,30,32]. While uncleaved pVII is detected in immature virus, only the cleaved pVII (pVII_N2_) is detected in mature virus [32,33].

In addition, the interaction of the cellular cullin-3 E3 ubiquitin ligase complex with the propeptide (amino acid 1–24 of pVII) increases the stability of pVII [34]. The propeptide sequence and the lysine residues at amino acids 26 and 27^th^ of pVII function co-dependently in providing stability to pVII [34].

The pVII localizes to different subcellular structures, including the nucleus, nucleolus and mitochondria [35,36]. The pVII is localized to the nucleus by active transport utilizing nuclear localization signals (NLSs) [36,37,38] and importin α/β and transportin nuclear import pathways. Although bioinformatic analysis of HAdV-5 pVII predicted amino acid 90–113 and amino acid 141–158 as potential NLSs, the revelation of the precise location of NLSs has been elusive. While analysis of the HAdV-5 mutant pVII protein suggested the presence of both classical (_99_KRRRRR_104_) and two overlapping potential bipartite NLSs at ^127^RARR^130^-X10-^141^RR^142^-X10-^153^RSRRR^157^) and non-classical (^188^RVPVRTRPPRN^198^) NLS, point mutations in these regions did not abolish the nuclear localization of pVII [37], indicating that the nature of NLS(s) utilized by pVII appear complex. Nevertheless, it appears that while uncleaved pVII specifically interacts with importin-α, importin-β, importin α-7 and transportin, the cleaved pVII specifically interacts only with transportin [37,38].

Interestingly, only uncleaved pVII localizes to the nucleolus due to the presence of nucleolar localization signals [36]. The pVII expressed by bovine adenovirus-3 (BAdV-3) also localizes to mitochondria using its own mitochondrial localization signal (MLS) located in the N-terminus amino acids 1–54 [35].

#### 2.1.2. Functions

The cleaved pVII associated with the viral genome mediates nuclear transport of the viral genome. Incoming viral DNA-protein core transports to the nuclear pore complex (NPC) with the help of host microtubule-dynein dependent mechanism [39,40] and docks at NPC by binding of N-terminus of Nup214 with hexon protein of partially disassembled virus [41]. After disassembly, nuclear transport of the viral genome appears to be mediated by genome-associated pVII import pathway utilizing multiple nuclear import receptors [37]. Thus, pVII appears to act as an adaptor for nuclear transport of the viral genome. A recent report indicates that Nup358 helps to form and increase different nuclear import receptors near NPC, allowing the adenovirus genome to exploit the pVII mediated nuclear import pathways [42].

A number of reports suggest that pVII interacts both with DNA and cellular proteins to regulate the viral life cycle. It is widely accepted that pVII is a functional analog of the cellular histone (H) [26,43]. H4 is one of the subunits of histone, which is acetylated and phosphorylated [44]. Although H4 subunit and pVII do not share much structural similarity, both proteins a) contain non-random distribution of basic residues [43], b) appear acetylated and phosphorylated [29,44] and contain a 1:1 protein–DNA mass ratio [43]. Interestingly, pVII functionally mimics cellular protamine [45], which is an arginine-rich nuclear protein that replaces histone during spermatogenesis for DNA condensation of the sperm head. Unlike protamine, protein VII interacts with nucleosomes but does not replace histones from nucleosomes [44].

The incoming cleaved pVII bound to AdV DNA plays an important role in preventing the induction of antiviral immune response, which appears essential for efficient viral replication in infected cells [29]. In response to inflammation, high mobility group B (HMGB) protein 1, a member of HMBG B group proteins, is released and acts as a danger signal to activate an immune response in the host cells [46,47]. Remarkably, cleaved pVII associated with the adenovirus DNA bind with HMGB1 in the nucleus and prevent its release from the cellular chromatin, thus helping in evading the induction of host cellular defense mechanisms [29]. Moreover, during the initial phase of the adenovirus infection, pVII masks the AdV DNA termini, preventing the recognition of AdV DNA termini by the DNA damage response sensor MRN (Mre11-Rad50-Nbs1) complex, thus helping in efficient viral DNA replication [48]. Similarly, pVII interacts with SPOC1 (survival-time-associated PHD protein in ovarian cancer 1), a chromatin-associated factor playing a role in DNA damage response and restricting adenoviral gene transcription in the early phase of infection and prevents the identification and detection of the viral dsDNA [49,50].

Recent reports have suggested that pVII may be involved indirectly in the early stages of virus–cell interaction [48,49]. Successful completion of AdV replication requires efficient entry and subsequent release of partially uncoated AdV from the endosomes. It is well established that endosomal acidification and disruption of the endosomal membrane by the lytic part (pVIn) of pVI help to release the partially uncoated adenovirus virion from the endosomes to the cytoplasm. An earlier study suggested that the 22nd amino acid of N-terminus cleaved pVII fragment (pVII_N2_) containing amino acids 14 to 24 appears to compete with the 23rd amino acid of N-terminus of cleaved pVI (pVI_N_) for interaction with the same binding sites of each hexon [32]. The vigorous competition between pVII (500–800 copies/virion) and pVI (360 copies/virion) for the same binding sites (720 sites/virion) on the hexon results in the availability of less abundant pVI to proteolytic cleavage by AdV protease, exposing N-terminal pVI lytic peptide (pVIn) for interaction with endosomal membranes leading to the successful endosomal escape of the adenovirus [50]. Inefficient escape of the adenovirus lacking pVII from endosome lead to the speculation that pVII may act directly in the early stages of infection or indirectly by altering interactions between core and capsid proteins [22]. Recent findings suggest that inefficient escape from the endosome of the adenovirus lacking pVII is due to the inability of AdV protease to cleave pVI, which remains hidden in the hexon cavity (due to availability of more binding sites on hexon), thus abandoning the availability of pVI lytic peptide in the endosome for interaction with endosomal membranes [50].

Adenoviral genome transcription is temporally regulated as different regions of the AdV genome transcribe at different times post-infection [4]. The adenoviral pVII is involved in the facilitation of the transcription of early genes of the adenovirus [51,52]. Once the pVII-DNA complex enters the nucleus, pVII remains associated with the viral DNA [51] and acts as a powerful transcription repressor [51,52]. During the early phase of infection, protein VII appears to associate with a cellular phosphoprotein 32 (pp32) and forms a complex with the viral chromatin [53]; however, p32 does not appear to remodel the adenovirus genome in vitro [54]. Interaction of pVII bound to the viral DNA with host factor SET/TAF-I (template activating factor) forms a DNA-pVII-TAF1 tertiary complex [55]. Formation of this complex leads to remodeling of the adenovirus genome, which facilitates and enhances the E1A transcription [55]. The E1A transcripts appear to release pVII from the genome [51,56], reverting the transcription repression. The release of pVII from the genome [56] and interaction of pVII with the N-terminus of E1A enhances the transcription of other early genes [51].

During adenoviral infection, pVII may prevent random deposition of the cellular histones on the newly replicated viral genome to avoid the negative effect on the transcription activity [55]. However, at late times post-viral infection, the interaction of the newly synthesized pVII complexed to the adenovirus DNAs with TAF-III (nucleophosmin/B23/NPM1) appears to remodel viral chromatin in infected cells [57]. Later, cellular zinc finger protein 622 (ZNF 622) appears to interact with pVII and TAF-III forming a trimeric complex, which limits pVII binding to the viral genome and hindering viral replication [58].

The AdV pVII is involved in condensing genomic DNA in AdV capsids [51]. An uncleaved pVII interacts with the AdV genome, forming complexes with DNA and histones [29], organizing the double-stranded DNA into condensed adenovirus core with about 180 nucleosomes [59]. This compacts 26–48 kb AdV genome in 90–100 nm icosahedral capsid and makes AdV core a pressurized and firm structure [60]. The basic nature of cleaved pVII is directly responsible for the rigidity and the pressure of the core and the stiffness of the capsid [61]. A recent report supports earlier proposed co-assembly mechanism of progeny virus formation [62] and proposes that, while the double-stranded genome is condensed by protein VII and other core proteins, the N-terminus of pVII (amino acid 1–24) acts as an anchor to assemble capsomers around it, which is cleaved off at maturation stage leaving condensed genomic core free of capsid [32]. Although pVII binds to ds DNA in a sequence-independent manner and can condense the viral genome [24,51], it is not essential for the viral DNA condensation in the viral capsid [22]. Moreover, pVII is not required for genome packaging or virus assembly [22], as speculated earlier based on the interaction of pVII with pVIa2 and p52/55k [63].

The pVII appears to be involved directly or indirectly in the proteolytic cleavage of some viral proteins [22]. The final step in the production of mature progeny virions is the AdV protease cleavage of precursors of AdV proteins VI, VII, VIII, IIIa, TP, X and 52/55K [16,30,64]. In the absence of pVII, proteolytic cleavage of some of these proteins, specifically pVI, appears defective, which has led to the suggestion that pVII may affect the proteolytic cleavage of pVI by altering the AdV protease activity [22].

Other reports suggest that pVII may facilitate efficient production of progeny virus by inhibiting apoptosis [35] or eliminating the inhibitory effects of a cellular protein [65]. The pVII localizes to mitochondria, which helps to inhibit apoptosis by retaining the mitochondrial Ca^2+^, increasing the mitochondrial ATP level and maintaining the membrane potential (MMP) of transfected cells [35]. Moreover, an un-cleaved pVII interacts with CCCH-type zinc finger protein named cellular Makorin ring finger protein 1 (MKRN1; E3 ubiquitin ligase) and together with unknown cellular factor enhances MKRN1 self -ubiquitination followed by proteasomal degradation of MKRN1 in infected cells [65], which may help in efficient viral production.

The pVII also has been reported to interact with pIVa2, p52/55 kDa [63], hexon [50], pV [66], and pIIIa [67], however the biological significance of these interactions is not clear yet.

### 2.2. Protein V (pV)

#### 2.2.1. General Characteristics

The protein V (pV), a unique protein encoded by only members of genus mAdV [11]. pV resemble cellular histones [59,68] and is highly basic in nature containing high arginine and lysine residues. pV [23,68]. The pV is the second most abundant core protein; however, variable relative concentrations of pV versus most abundant core protein pVII have been reported to be 1:6 and 1:3.5, respectively [24]. Each virion contains 148 ± 15 copies of pV [69]. Crystallographic analyses of the viral capsid suggest that pV has an extended structure with two short peptide helices (amino acids 208–219 and amino acids 259–271) [70]. Cross-linking studies involving virus particles suggest that pV appears to be in a soluble form in an equilibrium of monomer–dimer [68]. The pV is acetylated at its amino terminus [71] and is a target for host SUMOylation machinery, which appears to regulate the viral replication efficiency [72].

Once the infectious mAdV internalizes a target cell, uncoating the capsid takes place in a stepwise fashion [73]. Earlier reports suggested that incoming pV attached to DNA containing virus core [74] or associated with the cellular p32 [75] enters the nucleus. However, a recent report suggests that although pV is bound to the incoming viral genome, it dissociates from the virus partly at the entry of the AdV core in the cytoplasm and at the nuclear pore complex [59] without entering the nucleus. In contrast, newly synthesized pV actively transports to the nucleus with the help of its own nuclear localization signals (NLSs) by utilizing α/β nuclear import receptor-mediated nuclear transport machinery [76,77]. While a number of studies have reported the presence of both monopartite [76,77] and/or bipartite [76] NLSs, the location of NLSs and use of nuclear receptor for nuclear transport of pV may differ among pV encoded by different members of mAdV [38,76,77].

Efficient replication of some viruses, including AdV, induces alterations in the nucleolus [76,78,79], which requires viral proteins to localize to the nucleolus. Interestingly, pV also localizes to the nucleolus of both infected and transfected cells [76,77] though pV does not accumulate in the nucleolus [38]. Deletion mutant analyses suggested that pV encoded by different mAdVs contain two independent nucleolar localization motifs, which appear to be functionally redundant [76,77] and may utilize transportin for nucleolar localization [38]. Interestingly, the production of stable infectious virion requires the presence of both NoLSs [76]. While nucleolar localization of HAdV-5 pV induces translocation of B23.1 and nucleolin in transiently overexpressed transfected cells [76], the nucleolar localization of BAdV-3 pV does not alter the nucleolar distribution of B23.1 or nucleolin in infected/transfected cells [77]. Remarkably, pV encoded by mAdVs appears to contain both non-overlapping and overlapping NLS/NoLS [76,77].

#### 2.2.2. Functions

Earlier reports indicated that both pV and pVII might be involved in the translocation of the viral genome to the nucleus [37,74,75]. However, a number of observations suggest that pV may not be associated with the nuclear import of the viral DNA. First, the interaction of pV with pVII-DNA does not appear to be strong [74]. Second, pV seems to be fully dissociated from the adenoviral chromatin just before the nuclear localization of the viral genome [59]. Third, the viral genome is still translocated to the nucleus in pV deleted AdV suggesting that pV is not essential for the nuclear localization of the adenoviral genome [80].

In the absence of pV, AdV produces heat-labile progeny virions with altered morphology and infectivity [80,81], suggesting that pV may help in producing stable progeny virions. In fact, pV appears to act as a bridging factor between viral chromatin—viral core and between viral core—viral capsid by interacting with other viral proteins and DNA [66,68]. Both monomer and dimer of pV could be involved in these interactions in infected cells [68]. The basic amino acid-rich N-terminus of pV appears to interact at multiple sites with the viral DNA, which appear heat-stable [68]. The binding of pV to the viral DNA leaves several regions of pV available for binding to other viral proteins, such as pVII and Mu [66,68,75]. A recent report indicated that BAdV-3 pV interacts with a minor core protein, pIVa2, which may help to stabilize the bridge between viral DNA and viral core [82].

The pV interacts with other capsid proteins and acts as a bridge between the viral core and viral capsid [17,66,67,68,75]. The C-terminus of pV (amino acids 289–295) interacts with a minor capsid protein pVI (amino acids 103–115), making a bridge between the viral core and the capsid [70]. Additionally, pV interacts with the C-terminus of pVIII, another minor capsid protein [70]. Thus, interactions between pV, pVI and pVIII make a complex, which glues peripentonal hexons to an adjacent group of nine hexons [70]. However, the crystallographic structural analysis revealed that pV does not interact directly with hexons [70].

In addition to virion stabilization by bridging the viral DNA, viral core and viral capsid, pV seems to be an essential protein for adenoviral replication in primary cells, but not in cancer cells [80]. Unlike HAdV-5 [80], BAdV-3 pV appears essential for virus replication in both primary and continuous cell lines [81]. Moreover, deletion of BAdV-3 pV did not result in the introduction of compensatory mutations in pX/Mu or pVII [80,81]. While deletion of pV does not appear to affect the expression of early proteins, the expression of some late proteins is altered in infected cells suggesting that pV may be involved in the regulation of late gene expression [80,81].

The highly basic N-terminus of pV interacts with genomic DNA [70]. The pV linked with each unit of DNA-pVII hexamer [24,68,83] may be helping in the condensation of the viral genome [59]. Moreover, the interaction of pV with pIVa2 (role in DNA encapsidation) has suggested that pV may also play a role in the viral DNA encapsidation [82]. Similarly, the interaction of pV with nucleophosmin 1/NPM 1/B23.1 [84] and pVII [66] has led to speculation about its role in virus assembly and viral mRNA transcription, respectively.

Furthermore, pV interacts with nonstructural proteins 33K and 100K in infected cells [85], suggesting its potential role in other stages of the viral replication. pV also interacts with structural proteins penton base [86] and pIIIa [67], exhibiting the complexity of pV viral protein interactions.

### 2.3. Protein IVa2 (pIVa2)

#### 2.3.1. General Characteristics

The AdV protein IVa2 (pIVa2), encoded by the intermediate region of the AdV genome [11,14], is transcribed from complementary DNA strands by alternative splicing [87]. The expression of IVa2 mRNA appears to be upregulated by human antigen R (HuR) protein [88]. Initially, pIVa2 transcription initiation sites and cellular transcription repressor factor (RF) binding sites of pIVa2 are superimposed [89]. Due to the association of pIVa2 promoter sequence with transcription repression factor (RF), the expression of pIVa2 gets delayed compared to other early proteins except for pIX and E2 proteins [89]. However, once DNA replicates, the pIVa2 promoter gets activated, and pIVa2 transcription starts [90].

pIVa2 is expressed as 50 kDa, and a 40 kDa truncated protein in HAdV-5 infected cells [91,92]; however, only 50 kDa protein has been detected in BAdV-3 infected cells [82]. The pIVa2 is one of the core proteins [1,93] located interior to the viral capsid and is detected in both the assembly intermediates and mature virions in precursor form [94]. A mature adenoviral particle appears to contain 6–8 copies of unprocessed pIVa2 [95].

Efficient adenoviral replication requires the transport of the viral proteins to different cellular compartments, including the nucleus and nucleolus. The pIVa2 actively localizes to the nucleus using importin receptor of importin α/β nuclear import pathway and pIVa2 specific NLS [82,96]. Moreover, pIVa2 localizes to nucleolus using multiple functionally redundant NoLSs [82,96]. Interestingly, the location of pIVa2 NLS and NoLS encoded by different mAdVs does not appear to be co-linear [82,96].

#### 2.3.2. Functions

The AdV pIVa2, a multifunction protein, plays a significant role in different steps of the viral replication by interacting with the viral DNA and different viral/cellular proteins [96]. First, it acts as the transactivating factor, which activates the major late promoter (MLP) via downstream-binding element (DE), which contains two main binding sites called DE1 and DE2a and DE2b [97]. The protein pIVa2, a component of DEF-A and DEF-B, acts as a positive transcription factor and interacts with the DE element of MLP [98].

DEF-B (a homodimer of pIVa2) interacts with DE2b site, while a DEF-A (heterodimer of pIVa2 and p52/55K protein [99], pIVa2 and 22K [100] or pIVa2 and p33K [101] interacts with DE1 and DE2a sites [102]. Other than forming the DEF-A complex, the interaction between p52/55 kDa and pIVa2 seems required to avoid the premature formation of DEF-B, another transcription factor. Once pIVa2 activates MLP, it regulates the expression of most of the late AdV structural proteins [98,102]. Interestingly, mutant AdVs containing a deletion of pIVa2 or mutation of DE1 and DE2 site of MLP do not show a marked decrease in late protein expression [103,104]. Moreover, despite the similarity between DNA binding motifs of DE elements of MLP and AdV DNA packaging domain [105,106,107], mutation of the C-terminal DNA binding domain of pIVa2 significantly alters adenoviral DNA packaging with a moderate effect on late gene expression [108]. These observations suggest that pIVa2 may not play a critical role in the transactivation of MLP.

Second, pIVa2 functions in AdV DNA packaging. The pIVa2 alone as a homodimer or as a complex with the viral p22K [109,110] interacts with the CG nucleotides of A-repeat consensus sequence (5-TTTG-(N8)-CG-3) [105,111] of the adenovirus DNA packaging domain located at the left end of AdV DNA. In particular, p22K appears to help in precisely localizing pIVa2 to A-repeats by promoting interaction between two pIVa2 monomers [111]. The binding of more than one motifs of pIVa2 with the packaging domain could arrange pIVa2 as a multimeric structure on the packaging sequence [112].

Several observations suggest that pIVa2 appears to mimic an ATPase [104,113], which hydrolyze the ATP required for DNA packaging [114]. First, like ATPases, pIVa2 also contains Walker A and Walker B motifs, a characteristic feature of ATPases [113]. Second, secondary structure analysis of pIVa2 predicted similarity to the additional strand catalytic E (ASCE) class of ATPases [115]. Third, pIVa2 binds and hydrolyze ATP [113,116]. The presence of highly conserved Walker A and B motifs in pIV2 encoded by diverse AdVs [113] and non-viability of viruses containing the mutation of the conserved lysine in Walker A motif of pIVa2 [91] suggest that the function provided by Walker motifs is essential for the production of progeny virions. The Walker A and B motifs are involved in the binding and hydrolysis of ATP [113], thus providing the energy required for DNA packaging in preformed empty capsids. The ATPase activity of pIVa2 appears to be stimulated by the presence of protein p33K and the adenoviral genome [117]. Mutant AdV containing a mutation in Walker B motif of pIVa2 assembled into the empty capsid, which did not contain adenoviral DNA, suggesting that ATP hydrolyzing machinery of pIVa2 is essential for the insertion of the AdV DNA to the empty capsids [104]. Though earlier reports suggested an important role of pIVa2 in capsid assembly [118], the formation and detection of empty capsids in pIVa2 deleted AdV mutant suggests that pIVa2 does not play a role in AdV capsid assembly [104].

Many DNA viruses encode proteins forming dodecameric portal proteins, which help to insert viral DNA in empty capsids [119]. The packaging machine consists of a packaging ATPase (large terminase), a small terminase and portal protein(s) [120]. Though DNA viruses are known to encode portal protein (s) [119], the identity of such proteins is not known in AdV. Analysis of HAdV-5 E4 34K protein, including detection of interaction with packaging proteins, including IVa2, has led to the speculation that E4 34K may act as an AdV portal protein [121]. Detection of pIVa2 on a single unique vertex of mature AdV virion [95] and identification of pIVa2 and p22K complexes on adenoviral DNA packaging domain [109,122] has led to the speculation that pIVa2-p22K may act as a portal structure. Interestingly, adenoviral pIVa2-p33K-pDBP have also been detected at the unique vertex of AdV [123], leading to the speculation that this protein complex may play a role in the translocation of AdV DNA during virus assembly. Although the identity of portal protein [121] and small terminase [123] remains speculative, it is clear that pIVa2 serves as a packaging ATPase.

### 2.4. Adenovirus Protease (Adenain/AVP)

Adenovirus protease (AVP), a conserved endopeptidase encoded by AdVs during the late phase of the infection [31,124], is a highly basic protein [125], which represents a novel class of cysteine proteases [126]. It specifically recognizes sequence motifs, (M/I/L) XGX-G and (M/I/L) XGG-X (where X can be any amino acid) [15,127]. Interestingly, AVP appears phosphorylated [128]. About 7–50 copies of AVP are packaged in mature virions [69,129].

New AVP is synthesized in an inactive form [129], which becomes partially active after packaging in immature virion and binding to the viral DNA [64]. The viral DNA-bound AVP comes in contact with sliding pVI and cleaves it at N- and C-terminal releasing 11 amino acid peptides (amino acid 240–250) co-factor pVIc [130,131]. The binding of pVIc to AVP, bound to the viral DNA, activates the protease [130,131]. Finally, the adenoviral precursor proteins are located by sliding of AVP–pVIc complex along the viral DNA in the virions [132]. A recent study has shown that 11 amino acid pVIc acts as a molecular sled, which slides AVP along DNA [133].

AVP is essential for the viral maturation and production of infectious progeny virions [31] and proper disassembly/ release of the incoming virus particles in the cytoplasm [134]. AVP cleaves adenoviral precursor proteins (IIIa, VI, VII, VIII, Mu/X, and TP) in the virion [16]. In addition, a nonstructural protein 52K/55K required for genome packaging is also cleaved by AVP in the presence of DNA, recognizing both consensus and non-consensus protease cleavage sites [64]. Interestingly, AVP can also cleave proteins in the absence of two viral co-factors, namely viral DNA and pVIc [16,135,136]. AVP encoded by BAdV-3, HAdV-5, or porcine adenovirus-3 (PAdV-3) cleaves BAdV-3 100K in the cytoplasm of the transfected cells recognizing non-consensus (“FRASAF” and “IRAAGR”) viral protease cleavage sites, which is not essential for the replication of BAdV-3 [135]. Similarly, at late times post adenovirus infection, adenovirus protease cleaves cytokeratin 18 in the cytoplasm in the presence of actin [136].

The unique biology of AVP and, availability of crystal structures of the active and inactive form of AVP [137,138] has led to the development and evaluation of anti-adenoviral drugs [139].

### 2.5. Protein X (Mu)

Protein Mu (pMu), encoded by late adenoviral transcript, is a highly basic protein due to the high amount of arginine residues [140,141]. pMu contains non-overlapping nuclear and nucleolar localization signals [141]. In addition, pMu contains two functional consensus AVP cleavage site sequences [142], which are recognized by AVP to produce three fragments; (a) a hydrophobic N-terminus amino acid 1–31, (b) a basic middle part amino acids 32 to 50, and (c) C-terminal amino acids 51–79 [140]. About 100–300 copies of 19 amino acids short middle part (amino acids 32 to 50) are present in the mature virions [17].

Precursor pMu non-covalently binds viral DNA [143] and, together with other viral core proteins, plays a role in condensing the viral genome [27]. A highly conserved region in the C-terminus of the precursor pMu is involved in altering the accumulation of E2 proteins [141]. Interestingly, the addition of mature Mu peptide to liposome transfection reagent increased the transfection efficiency by 11% [144].

Cross-linking studies have revealed interactions between polypeptides V and Mu and VII-V-Mu proteins in the viral cores [66].

### 2.6. Terminal Protein

The terminal protein encoded by the E2B region of the adenoviral genome is a non-basic core protein [1,14], which is covalently bound to each 5’ terminus of the viral double-stranded DNA [145]. Thus, there are only two copies of terminal protein present in a virus particle [145]. Moreover, the precursor terminal protein contains two potential adenoviral protease cleavage sites [111,146]. Terminal protein serves as the primer for DNA replication and is one of the essential proteins for viral DNA replication [146,147]. In fact, terminal protein is required for efficient DNA replication initiation and to prevent false internal starts [146,148]. Additionally, the terminal protein appears to protect the adenoviral DNA from nucleases activity [146].

## 3. Summary

In summary, the last decade has elucidated the structure and function of some adenovirus core proteins in different stages of virus infection, including evading induction of innate immune response and initial stages of virus–cell interaction (Table 1). This could be due to the availability of more advanced techniques for exploring the structure of AdV and an interest in developing more efficient adenoviral vectors. Future studies aimed at determining the biological function of core protein interactions should help in exploring and providing more in-depth knowledge about core proteins in adenovirus biology.

## Figures and Tables

**Table 1:** Summary of *Mastadenovirus* core protein functions.

*Mastadenovirus* Protein	Functions
**VII**	Nuclear transport of the viral genome [37,42] Acts as the cellular histone [26,29,43,44] Functionally mimics protamine [45] Prevents induction of innate immune response [29] Prevents recognition of AdV DNA-by-DNA damage sensor MRN [48] Counteracts SPOC-1-mediated antiviral response [49] Is involved directly/indirectly in the endosomal escape of uncoated virions and pVI cleavage by protease [22,50] Facilitates early gene transcription [51,52,55,56] Prevents deposition of the cellular histones on newly replicated AdV genome [55] Remodels viral chromatin at late times post-infection [57] Hinders viral replication at late times post-infection [58] Condenses AdV genomic DNA [29,51,59,60,61] N-terminus of pVII may act as an anchor to assemble capsomers around the condensed AdV genome [32] Facilitates efficient viral production by inhibiting apoptosis [35] or inhibitory effect of the cellular protein [65]
**V**	Produces of stable progeny virions [81] Interacts with the viral DNA and other core proteins [66,68,75,82] ii) Interacts with other viral capsid proteins [17,66,67,68,70,75] May be required for expression of late gene expression [80,81] May be involved in AdV genome condensation [24,59,68,83] May be involved in mRNA transcription [66], DNA encapsidation [82] and/or virus assembly [84] Other roles in virus replication [67,85,86]
**IVa2**	Activates AdV major late promoter [97,98,99,100,101,102] Packages adenovirus DNA [109,110,111,112] Acts as DNA packaging ATPase [104,113,114,115,116,126] and involved in the insertion of the viral DNA in empty capsids [118]
**Protease**	Is essential for virus maturation and production of infectious progeny virion [31] Is esential for the proper release of the incoming uncoated virion to the cytoplasm [134] Cleaves precursor adenoviral proteins IIIA, VI, VIII, Mu/X, TP and 52K/55K in virion [16,64] Cleaves 100K in the cytoplasm of transfected cells [135]
**Mu/X**	Condenses AdV genome [27] Alters accumulation of E2 proteins [141] Is involved in increasing DNA transfection efficiency [143]
**Terminal Protein**	Acts as a primer for DNA replication [146,147] Protects AdV DNA from nuclease activity [146]

## Data Availability

No new data were created or analyzed in this study. Data sharing is not applicable to this article.
